# Intraspecific variation in responses to extreme and moderate temperature stress in the wild species, *Solanum carolinense* (Solanaceae)

**DOI:** 10.1093/aobpla/plae030

**Published:** 2024-05-21

**Authors:** Emma K Chandler, Steven E Travers

**Affiliations:** Department of Biological Sciences, North Dakota State University, Dept. 2715, PO Box 6050, Fargo, ND 58108-6050, USA; Department of Biological Sciences, North Dakota State University, Dept. 2715, PO Box 6050, Fargo, ND 58108-6050, USA

**Keywords:** Climate change, local adaptation, plants, pollen germination, *Solanum carolinense*, temperature tolerance

## Abstract

Adaptation or acclimation to local temperature regimes has often been used as a proxy for predicting how plant populations will respond to impending novel conditions driven by human-caused climate change. To understand how plants may successfully respond to increasing air temperatures (extreme and moderate) in the future, we explored how temperature tolerance traits differ in populations of *Solanum carolinense* from northern (MN) and southern (TX) regions of the continental USA in a two-experiment study. In the first experiment, we compared the heat and cold tolerance in vegetative (sporophyte) and reproductive (male gametophyte) traits. In the second experiment, we studied if long-term heat influences plant development by examining how development in moderate heat affected reproductive structures and reproductive success. We found that temperature sensitivity differed between southern populations, which regularly experience extreme heat, and northern populations which do not. In contrast to our expectations, northern populations appeared more heat-tolerant than southern populations for vegetative traits such as chlorophyll stability and reproductive traits such as pollen germination. Our results are consistent with a heat-avoidance, rather than tolerance mechanism to mitigate extreme heat during pollen germination. In the second experiment, plants developing under the moderate heat treatment had significantly smaller reproductive structures and reduced seed production (27% fewer seeds on average than in the control treatment). Reproductive structures that developed in moderate heat were also reduced in size, particularly in the northern populations relative to populations from the south. We conclude that rising temperatures have the potential to incur substantial negative consequences for the reproductive success of individuals in this species and that some populations already mitigate stressful temperature conditions through phenotypic plasticity.

## Teaser

Climate change is rapidly altering local temperature regimes with important biological impacts. We can gain insight into how plants specifically might respond to change in the future by studying how they have responded in the past. We explored how a single plant species that has grown both in warmer and cooler climates in the past responds to extreme temperatures. Our approach was to grow horsenettle (*Solanum carolinense* ) plants from Texas (South) and Minnesota (North) side by side and test for their tolerance to extreme high and low temperatures. Overall, we found that heat has definite negative effects but, surprisingly, northern plants were more thermotolerant in extreme temperatures (including heat). Southern plants, in contrast, may use avoidancestrategies in extreme heat. We conclude that populations of horsenettle have adapted ways to mitigate temperature stress, which may allow them to persist in the face of unprecedented global change.

## Introduction

Climate change is rapidly altering environmental conditions at regional and local scales, leading to shifts in temperature regimes, precipitation patterns, and the severity of weather events (Seneviratne *et al.* 2021). As a result, there is widespread interest in understanding how plants, a mostly sessile taxonomic group, will cope with these rapid changes (Doak and Morris 2010; Molina-Montenegro and Naya 2012; Valladares *et al.* 2014; Demarche *et al.* 2018). Plants respond to environmental change by tolerating the new conditions or avoidance through adaptation, shifting phenotypes with sufficient plasticity, or through escape by shifting ranges (Janzen 1967; Schlichting 1986; Molina-Montenegro and Naya 2012).

We can begin to understand the process of adaptation to extreme temperatures by examining how plants have already responded to variable climate conditions across their native range. Selective pressures are more likely to differ among distant populations of species with large ranges. Divergent selection in two regions can result in differing trait optima in the separate populations through local adaptation (Kawecki and Ebert 2004). In the case of distant populations experiencing different climates, the expectation is that genetic divergence would lead to plants better adapted to either warmer or colder extremes. In this scenario, climate change would force divergent evolution resulting in plant populations better adapted to heat. For example, Franks *et al.* (2007) found that mustard plants (*Brassica rapa*) grown from seeds collected after years of drought were better adapted to drought than plants from seeds collected prior to the drought, even when grown side by side.

Alternatively, plants may be phenotypically plastic in their response to environmental conditions, resulting in populations with different traits, but little genetic divergence (Nicotra *et al.* 2010; Stotz *et al.* 2021). In this scenario, plants from different regions with different climates would nonetheless be equally tolerant of temperature extremes when grown side by side in the same environment. A current area of interest is the extent to which phenotypic plasticity is adaptive or maladaptive in response to climate change (Ghalambor *et al.* 2007; Nicotra *et al.* 2010; Dupont *et al.* 2024).

It is likely that increasing mean, maximum and minimum temperatures associated with climate change will have negative effects on important fitness-related traits in plants. Based on the IPCC Sixth Assessment Report (Seneviratne *et al.* 2021), temperatures are changing at unprecedented rates. The National Climate Assessment (USGCRP 2018) reported that temperatures in the midwestern and southeastern USA have been steadily rising since the 1970s. Changes to temperature regimes are expected to ultimately lead to temperatures that are above what is currently optimal for plant cellular processes, especially those involved in reproductive success (Sato *et al.* 2006; Müller *et al.* 2016; Xu *et al.* 2017; Jiang *et al.* 2019). Researchers have experimentally established that development in moderately high temperatures negatively affects floral morphology (Charles and Harris 1972; Sato *et al.* 2006; Müller *et al.* 2016), ovule viability (Xu *et al.* 2017), pollen viability (Sato *et al.* 2006; Din *et al.* 2015; Müller *et al.* 2016; Xu *et al.* 2017; Poudyal *et al.* 2019), fruit set (Charles and Harris 1972; Sato *et al.* 2006; Din *et al.* 2015), and seed set (Din *et al.* 2015) in crop species. For many of these studies, heat was detrimental to development and reproduction. For example, Sato *et al.* (2006) found that elevated temperatures decreased fruit set and pollen viability as well as stamen height in tomatoes. Poudyal *et al.* (2019) found that pollen viability decreased in heat, but more tolerant tomato accessions had higher pollen germination than sensitive accessions. Xu *et al.* (2017) found that long-term moderate heat decreased pollen viability, pollen number, female fertility, and fruit set. Charles and Harris (1972) found that flower production, fruit set, fruit size, pollen germination, and distance between the stigma and antheridial cone all decreased at high temperatures in tomatoes. Lastly, Müller *et al.* (2016) found that long-term moderate heat resulted in floral deformations and low pollen viability in tomatoes. Researchers have repeatedly shown that heat has negative effects on reproductive traits, but few studies have examined how local adaptation and phenotypic plasticity may play a role in intraspecific variation in response to development in heat. We test the negative effects of heat on floral and reproductive characters to see if responses from genotypes that originate in different climates are consistent with expectations given the temperatures in the region of origin.

While previous studies have established that heat or temperature stress, in general, is detrimental to vegetative and reproductive traits in domesticated species, the question remains: can plants evolve tolerance or other strategies to mitigate temperature stress quickly enough to survive climate change? Selection for trait divergence might not occur if species can acclimate to novel temperatures through phenotypic plasticity (Nicotra *et al.* 2010; Stotz *et al.* 2021). However, acclimation would require a species to have evolved appropriate levels of phenotypic plasticity and the responses to cues that improve or maintain fitness (Schlichting and Pigliucci 1995; Ghalambor *et al.* 2007; Molina-Montenegro and Naya 2012). For example, Molina-Montenegro and Naya (2012) found that phenotypic plasticity in response to temperatures increased with latitude to match temperature regimes of the region of origin in *Taraxacum officinale*. In the case that a species cannot acclimate, populations in areas with temperature stress may face extirpation unless they have the potential to migrate to more favourable habitats. Lastly, variable local conditions introduce the possibility of divergent selection acting on the genetic diversity already within the population. Adaptation would involve a shift in tolerance through the evolution of traits that improve the chances of survival. For example, Driedonks *et al.* (2018) found that wild tomato accessions from populations at low elevations and high annual temperatures had high pollen viability under long-term moderate heat, leading the authors to conclude that those populations had locally adapted thermotolerance. Space-for-time substitutions are often used to study local adaptation to region-specific climate conditions as a proxy for how populations in areas of warming may respond to climate change.

To understand how plants will respond to a warming world, we tested for differences in temperature tolerance between plants from different thermal regimes. We examined how heat and cold stress affected traits in individuals of a widespread weed, *Solanum carolinense* (Solanaceae), from northern and southern regions of the continental USA. Our hypothesis was that plants from relatively warm environments (South) would be more tolerant to extreme heat and plants from relatively cold environments (North) would be more tolerant of extreme cold. Moreover, tolerance to heat would be negatively correlated with tolerance to cold due to tradeoffs in the mechanisms of thermotolerance.

We present the results of two experimental studies, where we sought to quantify heat and cold tolerance in plants from different latitudes and ultimately inform predictions of plant evolution in a warming environment (Nicotra *et al.* 2010; Demarche *et al.* 2018). The objective of Experiment 1 was to determine if there is evidence that local thermal conditions have divergently selected temperature tolerance traits between northern and southern latitudes by measuring the tolerance of plants to heat and cold extremes when grown in a common environment. The objective of Experiment 2 was to test for differences in tolerance specifically to heat by measuring developmental limitations when grown in hot environments. We hypothesized that southern populations of *S. carolinense* evolved greater heat tolerance compared to northern populations. Conversely, we expected—higher tolerance to extreme cold and lower tolerance to heat stress for plants from more northern populations. As a result, we expected northern plants would be less tolerant of heat when exposed to acute extremes of temperature (Experiment 1) and when grown in relatively high temperatures over a sustained period of time (Experiment 2).

## Methods

**Figure 1.**
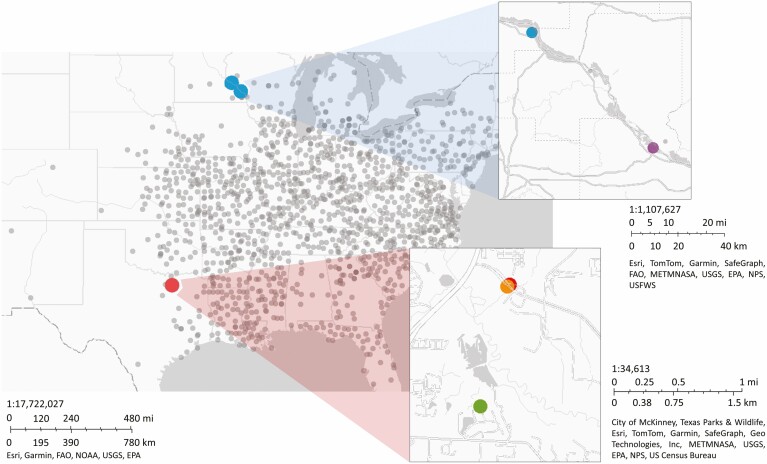
Map of the distribution of *Solanum carolinense* (EDDMapS 2022), northern and southern regions, and populations of origin for plants in this study. The populations Frontenac (top blow-up) and Prairie Island (top blow-up) were in the northern region and the populations Cemetery (bottom blow-up), Oil Patch (bottom blow-up), and Reserve (bottom blow-up) were located in the southern region.

*Solanum carolinense* L. (Solanaceae), also known as horsenettle, is a weedy, andromonoecious, perennial that originated in southeastern North America. As the genus suggests, *S. carolinense* is closely related to eggplant, tomatoes, and other common crops in *Solanum*. All other species in the Carolinense clade are neotropical, suggesting that this species likely arose through dispersal to North America and independent diversification (Wahlert *et al.* 2014). Once established in the southeast, *S. carolinense* utilized its natural adaptability and propensity to reproduce both sexually and asexually to expand its range north- and west-ward into colder and hotter environments (Fig. 1).

We collected 42 *Solanum carolinense* plants from two populations in Minnesota and 26 plants from three populations in Texas between October 2019 and August 2020 (Fig. 1). The Minnesota plants collectively will be referred to as the northern plants and include the populations ‘Prairie Island’ (44.07959 N, -91.684545 W) and ‘Frontenac’ (44.523056 N, -92.338611 W). Approximately 80 km separated the two populations (Fig. 1). In Houston County, MN where these plants were collected, the daily temperatures vary from a low of −34 °C to a high of 38°C over the course of the year. The Texas plants together will be referred to as the southern plants. All three Texas populations were located within a circle with a 1.5 km radius near McKinney TX (‘Oil Patch’: 33.173465 N, -96.615402 W; ‘Reserve’: 33.159962 N, -96.619011 W; and ‘Cemetery’: 33.173672 N, -96.615096 W). In Colin County TX, where these plants were collected, the daily temperatures vary from a low of −10 °C to a high of 45 °C over the course of the year (Supporting Information**—**Fig. S1).

We harvested plants by removing rhizomes (≥ 10 cm in length) from mature plants in the field and placing them in ziplock bags. Southern and northern rhizomes were collected on 25 October 2019 and 28 October 2019, respectively. We assumed that each rhizome represented a unique genotype (genet) because we maintained an interval of at least 1 m between collections. Other studies have assumed unique genotypes are found 1–2 m apart (Elle 1999; Wise *et al.* 2008). The sample sizes were 42 genets from the north and 26 genets from the south.

The field-collected rhizomes were shipped to Fargo, ND, and stored in a 4 °C refrigerator prior to a growth and dormancy period to establish an experimental population. After one week, the rhizomes were potted in 3.8 L containers with a ProMix BX standard potting mix and again stored in the 4 °C refrigerator. After eight weeks plants were placed in the greenhouse allowing above and belowground material to grow for six months. The above ground material was then cut, and the pots were again stored in a 4 °C refrigerator to induce a period of dormancy.

**Figure 2.**
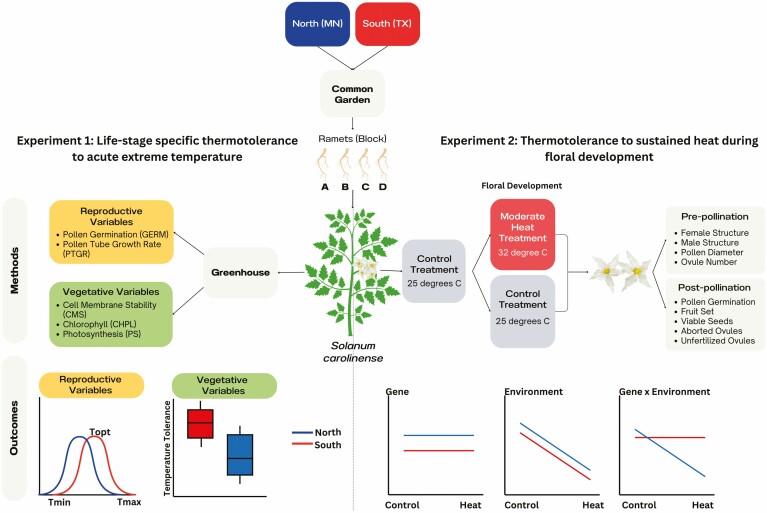
Conceptual diagram of methods and potential outcomes for Experiment 1 (left) and Experiment 2 (right). Experiment 1 examines responses to extreme temperature in vegetative traits using the relative structural damage to leaves in extreme hot and cold temperatures and reproductive variables using temperature performance curves of pollen performance measures. The outcomes illustrate the prediction that southern plants have a higher temperature tolerance to heat. Experiment 2 examines the response of reproductive traits to development in heat using morphological measurements and reproductive output for plants exposed to moderate heat vs controlled conditions. The outcomes represent potential interactions between treatments and regions of origin and their implications.

After the dormancy period (3 months), equal sections of rhizomes (at least 2 cm for thick rhizomes and increased lengths for thinner rhizomes) were cut into four equal-sized pieces. These were ultimately used to grow genetically identical plants (ramets) from each genet at different times (temporal blocks A, B, C and D; Fig. 2) because of a lack of sufficient space to grow them all at once. All ramets in block A were planted over the course of five weeks (10–12 plants per week) prior to the planting of the ramets in block B and so on. Of the ramets planted each week, half were from the southern region and half were from the northern region. Since we had a total of 26 genets from the south, we randomly selected 26 of the 42 genets from the northern populations using a random number generator to maintain a paired design. We tested a total of 26 genets from the northern and southern regions, with four ramets of each for a total of 208 plants. Each northern plant was paired with a southern plant spatially on the greenhouse benches. The rhizome pieces were originally placed in 3.8 cm diameter cone-shaped containers in the greenhouse. The plants were fertilized every other week with 10–10–10 fertilizer and transplanted to larger, 4.5 L containers when they outgrew the small cone-shaped containers.

### Experiment 1: Life-stage specific thermotolerance to acute extreme temperature

We grew plants from the north and south in a common garden to remove environmental effects and tested for differences in tolerance to acute exposure to extreme temperatures. Thermotolerant plants are expected to better survive maximum temperatures that could potentially damage plant tissues despite the short-term exposure to those temperatures. To assess the temperature tolerance limits on plants, we measured three vegetative variables (cell membrane stability (CMS), chlorophyll content (CHPL), and net photosynthetic rate (PS)) and two reproductive variables (the propensity for pollen grains to germinate (GERM) and the growth rate of pollen tubes (PTGR; Fig. 2)). We measured each variable on each plant for two temperature treatments, always relative to a control treatment: hot treatment (acronym preceded by ‘H’) and cold treatment (acronym preceded by ‘C’). These are standardized protocols for establishing thermotolerance in plants (Martineau *et al.* 1979; Gajanayake *et al.* 2011; Fang and To 2016). The temperatures were chosen, not to mimic the natural environment, but rather to represent intermediate temperatures that do not completely destroy plant tissues but instead allow a comparison between more tolerant and less tolerant individuals.

### Vegetative temperature tolerance variables.

MS was calculated according to the widely used protocols from Gajanayake *et al.* (2011) and Fang and To (2016). Ion leakage from leaf material exposed to either heat (55 °C water bath for ten minutes) or cold (−18 °C) was measured using an electrical conductivity probe and compared to the conductivity of leaf material in control (27 °C) and maximum damage (98 °C) treatments. The heat treatment (55 °C) used was based on the protocol from Fang and To (2016) while the cold treatment (−18 °C) was selected based on the standard methods used in Mishra *et al.* (2011). Difference in chlorophyll content of leaves was estimated, as in Gitelson *et al.*, (1998) for material exposed to either a hot temperature treatment (60 °C for 1 hr) or a cold temperature treatment (4 °C for 1 h followed by −18°C for 1 hr) using a chlorophyll metre (Opti-Sciences CCM-300). The chlorophyll meter measures the fluorescence emitted at 735 nm/700 nm for a constant leaf area. Chlorophyll content before and after treatments was used to estimate the difference in chlorophyll content in mg/m^2^. The third vegetative variable (PS) was a measure of the effects of temperature treatments on the photosynthetic capabilities of leaves. PS was estimated as the ratio of net photosynthetic rates after a temperature treatment compared to before the treatment (HPS: 33 °C, CPS: 10 °C for 48 hr) based on leaf measurements made with a Licor-6200 field photosynthesis system (LiCor, Lincoln, NE, USA). Temperature treatments were again based on standard values used in thermotolerance studies (e.g. Xu *et al.* 2014; Zhu *et al.* 2018; Poudyal *et al.* 2019; Sherzod *et al.* 2019) and the temperature limits of the environmental chambers used for this study.

#### Reproductive temperature tolerance variables.

We focused on two pollen traits as reproductive variables for estimates of male thermotolerance during the reproductive stage: (i) the propensity for pollen grains to germinate (GERM) and (ii) the growth rate of pollen tubes while exposed to a range of temperatures (PTGR). We paired measurements of pollen traits from plants in the north and south by sampling mature anthers of plants flowering simultaneously. Pollen germination at extreme temperatures was measured following a protocol from Kakani *et al.* (2002). Pollen from one flower per plant was dispersed over five petri dishes containing the mixture described in Reddy and Kakani (2007; 3% Bacto-Agar based growth medium (sucrose, Ca(NO_3_)_2_, MgSO_4_, KNO_3_, H_3_BO_3_)). The dusted plates were each placed at one of the five temperature treatments (10 °C, 20 °C, 25 °C, 30 °C, 40 °C) for 16 hr based on previous studies (Kakani *et al.* 2002; Reddy and Kakani 2007; Singh *et al.* 2008; Fang and To 2016). Four pictures of each plate were taken using a compound microscope (Leica DM500 microscope, Leica ICC50 HD camera) and the LAS EZ 2.1.0 software. We measured pollen germination (Germ) for each plate by counting the number of pollen grains per image that had produced pollen tubes and dividing that count by the total number of pollen grains observed. Pollen tube growth rate (PTGR) was calculated by dividing the length of the 20 longest pollen tubes by the time allowed for growth (16 hr). Tube lengths were measured with ImageJ (Schneider *et al.* 2012). Detailed methods are provided in the Supporting Information. At the end of Experiment 1, each experimental plant was cut back to soil level and stored at 4 °C for 3–9 months.

### Experiment 2: Thermotolerance to sustained heat during floral development

In this experiment, we tested for differences in temperature tolerance limits and responses to heat throughout the duration of floral development. Altogether, we examined how the development of reproductive structures in heat affects the pre-pollination morphology including the length of style, stigma, and anthers, the number of ovules, and the size of pollen grains and post-pollination processes including fruit set, seed set, seed condition, and pollen germination rates.

In contrast to Experiment 1, in Experiment 2 we tested for differences in thermotolerance between northern and southern plants by measuring the responses of floral development, reproduction and fruit development to heat sustained over a longer period of time. The rationale for this experimental design is that the vulnerability of plants to relatively high temperatures may be more nuanced than complete tissue or cellular breakdown (as tested in Experiment 1). Instead, a thermotolerant plant may be one that can continue to develop normal tissues (e.g. flowers) over a sustained period when exposed to relatively high temperatures.

After Experiment 1, we removed the aboveground portions of each plant and stored them at 4 °C for a final dormancy period of 3–9 months (depending on Experiment 1 temporal blocking). Two ramets of all 26 genets from the north and south were placed in a randomized grid pattern in a growth chamber (Conviron PGC-FLEX). Due to space constraints in the environmental chambers, only A and B ramets were grown initially. Ramets C and D were placed in the chambers six months later. For initial growth, all plants were exposed to ‘control’ conditions (25 °C day/25 °C night; fluorescent and incandescent lighting for 14 hr per day). Plants were fertilized once every two weeks with a high phosphorus fertilizer (12–55–6) to promote flower production (Super Bloom, Scotts).

Upon flowering, two ramets per genet were randomly assigned to the control conditions (25 °C day/25 °C night; 14 hr/10 hr) and two to the heat treatment conditions (32 °C day/25 °C night; 14 hr/10 hr; Fig. 2). By necessity, these were in different Conviron chamber models (control: Conviron PGC-FLEX; heat treatment: Conviron E7/2). Plants were watered daily. Subsequent flowers and fruits developed at either elevated temperatures (32 °C) or control temperatures (25 °C). The moderate heat treatment of 32 °C was selected based on the temperatures used in previous studies with moderate heat treatments in tomatoes and to mimic temperatures in the warmer end of the range that populations of this species might encounter (Sato, Kamiyama *et al.* 2006; Müller *et al.* 2016; Xu *et al.* 2017). The control treatment of 25 °C is halfway between the average daily temperatures for MN (22 °C) and TX (29 °C).

#### Pre-pollination phase variables.

The first three hermaphroditic flowers per plant, that developed in the respective treatments, were collected in ethanol and used for flower morphology measurements, ovule counts and pollen size measurements. Floral morphology traits, female structure (length of the style and stigma) and male structure (length of one anther), were measured under a dissecting scope. The number of ovules in each ovary was counted following a modified staining protocol adapted from Diaz and Macnair (1999). Pollen diameters of approximately 100 grains for one flower per genet were measured with the use of a microscope (Axio Scope A.1 Carl Zeiss, Germany) at 400x total magnification and the ‘circle diameter’ measurement tool on the Zen 3.1 software.

#### Post-pollination phase variables.

We pollinated three additional hermaphroditic flowers and collected the pollen of one more flower to measure subsequent female and male reproductive traits. Mature flowers were pollinated with a mix of pollen from 2 to 5 flowers (depending on the number of plants flowering at the time) that developed in the control conditions, with at least one flower from a southern plant and one flower from a northern plant represented in the pollen mix. Pollinations were accomplished by applying a mixture of pollen on the stigma with a dissection probe. Each pollinated flower was labelled with a jewellery tag. After pollination, the plants remained in their respective treatments for one week before we moved them into a greenhouse for the remainder of fruit maturation (Average daily temperatures 25.1 °C day/21.3 °C night). Once fruits were ripe (at least one month old), they were harvested. We measured fruit set (number of fruits produced/three flowers pollinated) and the seed set. The number of viable seeds, aborted seeds, and unfertilized ovules were counted under a dissecting scope. Seeds that were visibly flat were considered aborted. Small black or brown spots visible under a dissecting microscope were considered unfertilized ovules.

*In-vitro* pollen germination at 40 °C was used as a proxy for male reproductive success in high temperatures. Thus, we used 40 °C to distinguish between thermotolerant and non-thermotolerant genotypes. As described in Experiment 1, pollen from each plant was dispersed over a petri dish containing 3% Bacto-Agar based growth medium (sucrose, Ca(NO_3_)_2_, MgSO_4_, KNO_3_, H_3_BO_3_) and incubated at 40 °C for 16 hr. Four pictures of each plate were taken using a compound microscope (Leica DM500 microscope, Leica ICC50 HD camera) and the LAS EZ 2.1.0 software. We measured pollen germination (Germ) for each plate by counting the number of pollen grains per image that had produced pollen tubes and dividing that by the total number of pollen grains observed.

## Data Analysis

### Experiment 1: Life-stage specific responses to extreme temperature

To analyse differences in vegetative traits between plant origins and among genets, we fit six separate linear mixed effects models (one for each variable) using the lmer function from the *lme4* package (Kuznetsova *et al.* 2017). Region (north vs. south) was considered the fixed effect and the temporal block (A, B, C and D) and genet were random intercepts. Since there was a significant block effect in some of the variables, we compared plants from the north and south within blocks using a paired *t*-test (function *t*.test).

For the reproductive variables (GERM, PTGR), we fit quadratic temperature performance curves, determined using model selection (Supporting Information**>—**Fig. S2), to the multiple temperature measurements taken for each plant that flowered using the nls.multstart function in the *rTPC* package (Padfield and O’Sullivan 2021). From the quadratic curves of each plant that flowered, we extracted three key values for both pollen germination and pollen tube growth rate: the temperature minimum, temperature optimum, and temperature maximum. We then used the key values in six separate linear mixed effects models with region and population as fixed effects and genet as a random effect to determine if the response curves differed between regions. One outlier was identified using the Grubbs’ test for outliers, grubbs.test function in the *outliers* package (Komsta 2011), and subsequently dropped from the analysis.

We used Pearson’s method for correlation analysis (function cor) to identify associations between vegetative and reproductive variables. The Holm–Bonferroni method (function p.adjust) was used to adjust *P*-values to account for multiple correlations (Holm 1979). All data were analysed in R 4.1.2 (R Core Team 2020).

### Experiment 2: The effect of heat during development on reproductive traits

All pre- and post-pollination traits were analysed with eight separate mixed-effects models using the *lme4* package (Bates *et al.* 2015). The general structure for the models was region, treatment, the interaction of region and treatment, and temporal block (January vs June) as fixed effects and genet as the random intercept. The nuances of each individual model are described below. We used general linear mixed effects models (*lme4*; function lmer) for male and female structure lengths. Since we collected data for the mean pollen diameter of one flower per block per genet and several genets only had one ramet (block) that flowered, we omitted genet as a random effect to avoid overfitting the data and used a general linear model (function lm). We used generalized mixed effects models (*lme4*; function glmer) with a Poisson distribution for all count data, which included counts of ovules, viable seeds, unfertilized ovules, and aborted seeds. We tested for overdispersion using the *DHARMa* package (Hartig and Lohse 2022). Since data of pollen germination at 40 °C were proportions, we used a generalized linear model (function glm) with a binomial distribution for analysis. Since the model including genet as a random effect overfit the pollen germination data, we decided to drop genet. To examine significant interactions between region and treatment, we did a post-hoc analysis using the *emmeans* package (Lenth 2023; function emmeans). We conducted a correlation analysis for the mean male and female structures (function cor.test). The fruit set was analysed using a chi-squared test (function chisq.test).

## Results

### Experiment 1: Life-stage specific thermotolerance to acute extreme temperature

#### Vegetative traits.

Of the six vegetative traits measured in this experiment and modelled independently, four differed significantly between regions. In extreme heat (HCHPL) and cold (CCHPL), northern plants retained chlorophyll content more effectively than southern plants (Table 1, Supporting Information**—**Fig. S3). The chlorophyll content of northern plants was 5% and 19% higher than southern plants for the heat and cold treatments respectively. There was no significant difference between regions for cell membrane stability in extreme heat, but the cell membrane of southern plants was more stable than northern plants in extreme cold (Table 1). There was a significant block effect for both CCMS (likelihood ratio = 15.342, *P* < 0.001) and HCMS (likelihood ratio = 4.496, *P* = 0.034; Supporting Information**—**Table S1). Southern plants had higher CCMS than northern plants in blocks B (*t* = 2.190, *P* = 0.040) and C (*t* = 2.073, *P* = 0.049), but generally CCMS decreased in all plants, regardless of region, with later Blocks (Supporting Information**—**Fig. S4). Northern plants had a higher HCMS than southern plants (*t* = −2.910, *P* = 0.015) in block A, but this difference degraded in the later blocks (Supporting Information**—**Fig. S4 and Table S3). Lastly, there was a significant regional effect for changes in photosynthetic rate in extreme heat (Table 1). Southern plants maintained a photosynthetic rate closer to the initial measurement after the heat treatment. There were no regional or block effects on photosynthetic rate in response to cold.

**Table 1.** Vegetative and reproductive temperature tolerance results from mixed effects linear models with the fixed effect region (north vs south) and population and the random effects genet and block (omitted for reproductive variables). Due to overfitting the model, genet was included as a fixed effect for CCMS, HPS, and Tmin PTGR. Statistics in the table include the degrees of freedom (dF: numerator, denominator), *F* statistic (*F*), and *P*-value (*P*) estimated using a type III ANOVA with Satterthwaite’s method. Population and random effect statistical values reported in the Supporting Information**—**Table S1, as well as results from a mixed model using only control values (Supporting Information**—**Table S3).

Variable		Region				
		Expected	Observed	dF	F	P
Vegetative	Cell membrane stability (Heat)	S > N	-	1, 46	0.706	0.405
	Cell membrane stability (cold)	N > S	S > N	**1, 188**	**10.358**	**0.002**
	Chlorophyll content (heat)	S > N	N > S	**1, 48**	**4.166**	**0.047**
	Chlorophyll content (cold)	N > S	N > S	**1,188**	**104.054**	**<0.001**
	Photosynthetic rate (heat)	S > N	S > N	**1,47**	**5.614**	**0.022**
	Photosynthetic rate (cold)	N > S	-	1, 33	1.966	0.170
Reproductive	Pollen germination (Tmax)	S > N	N > S	**1,52**	**5.937**	**0.018**
	Pollen germination (Topt)	S > N	N > S	**1,52**	**6.684**	**0.012**
	Pollen germination (Tmin)^*^	S > N	-	1,41	0.030	0.865
	Pollen tube growth rate (Tmax)	S > N	-	1,54	0.037	0.848
	Pollen tube growth rate (Topt)	S > N	-	1,56	0.042	0.839
	Pollen tube growth rate (Tmin)	S > N	-	1,57	0.923	0.341

^*^Outlier removed. Bolded values: statistically significant (*α* = 0.05).

#### Reproductive traits.

**Figure 3.**
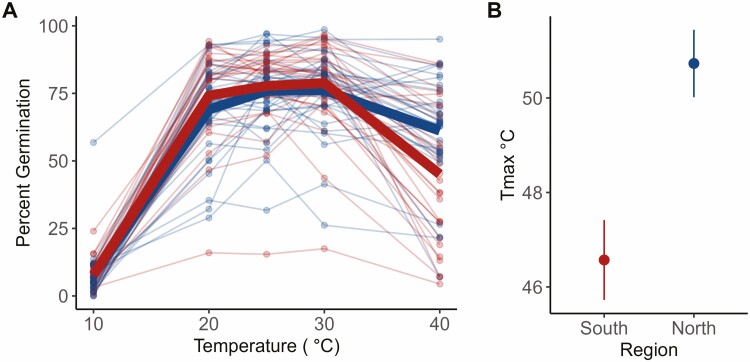
Pollen germination results from Experiment 2. (A) Percent pollen germination per genet (points) and regional mean (bolded lines: blue = northern, red = southern). (B) Mean (± se) Tmax for northern (blue) and southern (red) genets. Tmax is the upper x-intercept of the quadratic fit for temperature performance curves for each individual (i.e. the highest temperature predicted with pollen germination). For colour figure refer to online version.

There was a significant difference between regions for Tmax (Fig. 3, Table 1) and Topt (Table 1) in pollen germination. Pollen from plants from the north were more likely to germinate at high temperatures (Tmax) and had higher thermal optima (Topt) than pollen from plants from the south. There was no significant difference between the two regions for Tmin. One outlier was identified using the Grubbs’ test for outliers and subsequently dropped from the analysis. There were no significant differences in pollen tube growth rate between plants from the north and south for Tmax, Topt or Tmin.

#### Vegetative and reproductive tolerance correlations.

After a Holm–Bonferroni correction for multiple correlations, there were no significant correlations between the vegetative variables and any reproductive and vegetative variables. However, there were two significant correlation coefficients between reproductive variables (Supporting Information**—**Fig. S5 and Table S4). Tmax and Tmin of pollen tube growth rates were positively correlated (*r* = 0.46). There was also a significant correlation between Tmax for pollen tube growth rate and for pollen germination (*r* = 0.3).

### Experiment 2: Thermotolerance to sustained heat during floral development

#### Pre-pollination.

**Figure 4.**
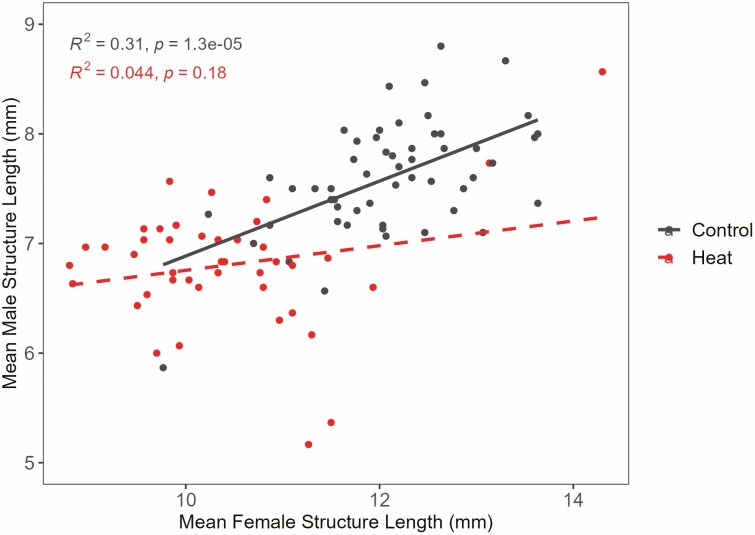
Correlation of the male and female structure length averaged across genets in Experiment 1.

**Figure 5.**
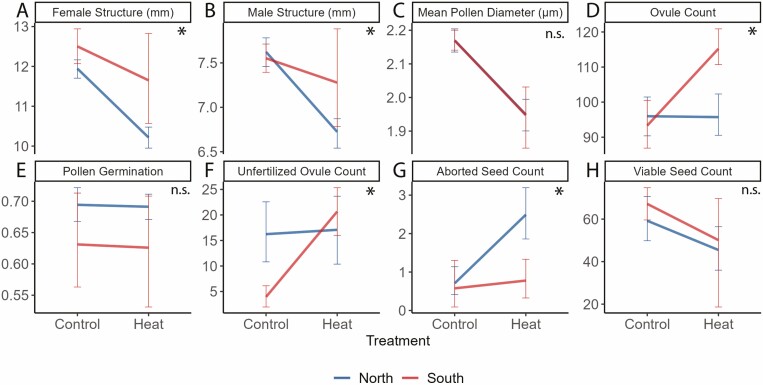
Interactions in Experiment 2 between heat treatment (heat and control) and region (North; blue and South; red). Error bars indicate the standard error and an asterisk indicate at least one significant interaction. For colour figure refer to online version.

We found that long-term moderate heat negatively impacted the female structure, male structure, pollen grain diameter, and ovule number (Table 2, Supporting Information**—**Fig. S5). On average, flowers that developed in the heat treatment had smaller floral structures. Female structure size decreased by 14% and male structure size decreased by 13% in long-term moderate heat conditions relative to the control (Table 2). Female structure length also differed by region of origin. Plants from Texas on average had 4% longer female structures than plants from Minnesota (Table 2). The relationship between male and female structure lengths changed with the development of heat (Fig. 4). Mean male structure length and female structure length were positively correlated in the control treatment, but not in the heat treatment (Supporting Information**—**Fig. S5). Development in heat increased the average number of ovules by approximately 1 ovule and reduced pollen size by 10%. Neither ovule count nor pollen size differed by region. We found significant interactions between treatment and region in the female structure, male structure and ovule number (Table 2; Fig. 5).

**Table 2.** ANOVA results with the fixed effects temperature treatment (control and heat), region of origin (north and south), the interaction between treatment and region, population, and temporal block (January and June). Genet was included as a random effect. Genet and population were excluded in the model for pollen germination at 40 °C due to overfitting the model. Results of temporal blocking and population in Supporting Information**—**Table S5.

Variable	Treatment			Region		Treatment:region	
	dF	*Χ* ^2^	*P*	*Χ* ^2^	*P*	*Χ* ^2^	*P*
Female structure (mm)	1	**242.98**	**<0.001**	**3.86**	**0.049**	**7.42**	**0.006**
Male structure (mm)	1	**205.84**	**<0.001**	**28.20**	**<0.001**	**9.45**	**0.002**
Ovule number	1	**10.50**	**0.001**	**10.95**	**<0.001**	**37.89**	**<0.001**
Pollen grain Size (μm) ^*^	1	**86.17**	**<0.001**	3.15	0.076	0.24	0.627
Pollen germination (40 °C)	1	0.00	0.968	0.307	0.579	0.00	0.997
Viable seed	1	**165.37**	**<0.001**	0.003	0.955	0.01	0.914
Unfertilized ovules	1	**89.48**	**<0.001**	0.14	0.710	**56.69**	**<0.001**
Aborted seeds	1	**23.28**	**<0.001**	1.39	0.239	**11.41**	**<0.001**

^*^Model excluded genet random effect to avoid overfitting model. Bolded values: statistically significant (*α* = 0.05).

#### Post-pollination.

Pollen development in long-term moderate heat did not affect germination at high temperatures and germination did not differ between regions (Table 2; Supporting Information**—**Fig. S6). Fruit set was also not affected by the heat treatment. The number of viable seeds was affected by heat (Table 2; Supporting Information**—**Fig. S6) and on average heat decreased seed set by 16 seeds. The number of unfertilized ovules increased by six in the heat treatment compared to the control and the number of aborted seeds increased by about 1.64 seeds on average (Table 2; Supporting Information**—**Fig. S6). We note here that the average number of aborted seeds in the control group was relatively low with an average number of 0.63 seeds. The number of unfertilized ovules did differ by region. There was a significant interaction between the treatment and region for the number of unfertilized ovules and aborted seeds (Table 2; Fig. 5).

Post-hoc comparisons of all significant interactions revealed that comparisons of the heat and control treatments were generally statistically significant (Table 3). In comparisons between control and heat treatments for northern plants, all except the interaction for ovule number was significant. Similarly, comparisons for the heat and control treatments for plants from the south were significant for all variables except aborted seeds. In the control treatment, northern and southern plants did not differ for any of the variables. In the heat treatment, northern and southern plants differed for female structure length and aborted seeds (Table 3). The comparisons of plants from the north in heat vs. south in the control were statistically significant for all variables except ovule number. Ovule number only varied for the two treatment groups in southern plants (Table 3; Fig. 5).

**Table 3.** *P*-values for post-hoc comparisons for significant interactions of treatment and region. The control and heat treatments are denoted with C and H, respectively. The letters N and S indicate plants from the north and south, respectively.

Variables	CN-HN	CN-CS	CN-HS	HN-CS	HN-HS	CS-HS
Female structure	***P* < 0.0001**	0.2051	0.3654	***P* < 0.0001**	**0.0011**	**0.0011**
Male structure	***P* < 0.0001**	0.3403	0.2676	***P* < 0.0001**	**0.0002**	**0.0027**
Ovule number	1.0000	0.1096	0.3830	0.1131	0.3945	***P* < 0.0001**
Unfertilized ovules	**<0.0001**	0.020	**0.0076**	**0.0004**	0.1109	**<0.0001**
Aborted seeds	**<0.0001**	0.8190	0.2308	**0.0006**	**0.0026**	0.3285

Bolded values: statistically significant (*α* = 0.05).

## Discussion

The results of the combined experiments we present here indicate that plants from northern and southern populations differ in their responses to both moderate and extreme temperature conditions. We found evidence that regional differences in *Solanum carolinense* have evolved over time based on the divergent patterns of tolerance to extreme temperatures (Table 1). Contrary to our expectations, we found that in multiple life stages, northern plants were more tolerant of extreme heat than are southern plants and southern plants were more tolerant of cold in terms of membrane stability. We also found that moderate sustained heat has a negative influence on floral development and reproduction, suggesting that phenotypic plasticity plays an important role in how plants respond to heat.

### Experiment 1: Regional differences in response to temperature extremes

Cell membranes from northern plants were more tolerant of extreme heat, while the cell membranes of southern plants were more stable in extreme cold. However, responses to extreme temperatures were not consistent and depended on when plants were grown in the greenhouse (temporal block). There was a significant temporal block effect for CMS in both the heat and cold treatment. The block effect on CMS may be due to the capacity of *S. carolinense* to induce temperature tolerance and acclimate to environmental conditions (Clarke *et al.* 2004). Temperatures in the greenhouse progressively rose throughout the spring and summer (Supporting Information**—**Fig. S7), potentially inducing heat tolerance and pre-conditioning plants for the temperature manipulations. Thus, a caveat of our study is that the temporal block limits the inferences we can make about region-specific responses to temperature stress.

Plants from the north had more stable chlorophyll content in both the hot (HCHPL) and cold treatments (CCHPL; Table 1). The capacity of chloroplasts in northern plants to outperform southern plants in both extreme cold and heat might be due to northern plants experiencing and adapting to a larger range of temperatures. Between 2018 and 2021, growing season temperatures (March to September) ranged from −28 °C to 34 °C (62 °C difference) in Houston County, MN, while temperatures ranged from −7 °C to 42 °C (49 °C difference) in Collin County, TX. If tolerance to extreme cold yields general physiological tolerance to any temperature extremes, northern plants, where wintering rhizomes remain in frozen ground for months, should be more tolerant than southern plants. Heat shock proteins, which play an important role in maintaining tolerance to heat in plant cells (Heckathorn *et al.* 1998; Feder and Hofmann 1999; Frank *et al.* 2009), can also confer tolerance to cold by stabilizing protein configurations and functions in cells at all stressful temperatures (Neta-Sharir *et al.* 2005). Therefore, selection for extreme temperature tolerance may be more common in northern latitudes. Our results suggest that increased thermal tolerance applies to chloroplasts in the north but not to cell membranes.

The results from reproductive trait comparisons also countered our expectations for the direction of temperature tolerance. Pollen from the north had a higher propensity to produce pollen tubes (Germ) at high temperatures than their southern counterparts (Table 1, Fig. 3). One possible explanation for these results is that there is an avoidance strategy in southern populations where maximum summer temperatures can reach over 38 °C consistently. Under these conditions, there could be a selective advantage to pollen remaining dormant rather than germinating at high temperatures. In contrast, there may be no selection for high-temperature dormancy—in the north. This explanation is supported by a theory regarding pollen dormancy developed by Rutley *et al.* (2022). They proposed the ‘two baskets model’ categorizing pollen, stating that there are active (high-ROS) and backup (low-ROS) subpopulations of pollen within anthers of flowering species. Active pollen readily germinates and has a fast metabolism, increasing pollen tube growth rates, and typically outcompetes the smaller, partially dehydrated backup pollen. The two subpopulations of pollen are adaptive under different conditions. In stressful environments, such as extreme heat or drought, asynchrony in pollen germination permits some pollen to remain dormant and grow pollen tubes later in more favourable conditions. In favourable conditions, active pollen tubes grow faster and are more likely to fertilize ovules than backup pollen. While the two-pollen system has not been established in *Solanum carolinense*, there have been studies demonstrating these two pollen types occur in *Solanum lycopersicum*, tomato (Jegadeesan *et al.* 2018; Keller and Simm 2018; Luria *et al.* 2019).

Predictions about how species will be affected by climate change can be improved with a better understanding of how different populations of the same species differ in their responses to heat. With climate change, plants in Minnesota are predicted to experience conditions that more closely resemble the current climate in Texas, including both higher average temperatures in summer months and higher maximum daily temperatures (IPCC 2014). Growing seasons are already lengthening in more northern latitudes (Badh *et al.* 2009; Dunnell and Travers 2011). Given the responses to heat by plants in our experiments in the form of relatively moderate heat (32 °C) during floral development, pollen tube growth and fruit maturation and extreme heat (40–60 °C) in acute doses, we suggest that plants in the two regions we studied have evolved some differences that represent differing strategies for surviving thermal stress.

There was little evidence that southern plants have evolved greater cellular tolerances to extreme heat despite growing in an environment that can have daily maximum temperatures above 40.5 °C. The stability of chlorophyll levels and pollen germination capabilities were reduced after exposure to extreme heat in southern relative to northern plants (Table 1, Supporting Information**—**Fig. S3). If plants in the south have shifted to an avoidance strategy where the temperature extremes of summer months are avoided by dormancy of pollen or flowering patterns shifted earlier or later, then selection for tolerance of high heat may not occur. In contrast, northern plants that experience relatively short seasons when growth and flowering are possible will need to flower and develop fruit during the hottest times of the year to produce viable seeds. However, as climate change leads to longer, hotter growing seasons, our expectation is that an upper limit to tolerance of heat will ultimately lead to different patterns in phenology and perhaps dormancy. Thus, the evolutionary consequences of climate change for flowering plants are likely to be a complex shift in phenology and physiological patterns.

### Experiment 2: Reproductive responses to moderate heat

As in other studies (Müller *et al.* 2016; Fahad *et al.* 2018), we found that exposure to higher temperatures during plant and floral growth led to negative effects on traits tied to successful reproduction. In Experiment 2, where plants were exposed to moderate heat (32 °C) and control conditions (25 °C) during floral development, there was a significant treatment effect on seven of the eight characteristics we measured (Table 2, Supporting Information**—**Fig. S6) including floral morphology measurements, pollen size and ovule fate (viable, aborted and unfertilized).

Male and female structures were smaller in the heat treatment compared to the control and more pronounced in plants from northern populations relative to southern populations (Table 2, Fig. 5). We did not specifically measure herkogamy, but the differing style lengths we observed could have implications for pollen competition (Ramesha *et al.* 2011) and the position of anthers relative to the stigma could affect the receipt of outcross versus self-pollen from pollinators. We found that the correlation between the length of male and female reproductive structures breaks down in heat (Fig. 4) suggesting that the fundamental proportions of floral structures are disrupted. Several other studies found that heat affects the floral structures in other taxa (Charles and Harris 1972; Sato *et al.* 2006; Müller *et al.* 2016), but not necessarily in the same way (Lyrene 1994). Muller *et al.* (2016) found anther deformations when tomato flowers developed in moderate heat (32 °C/26 °C). Charles and Harris (1972) found that as temperatures increased, the distance between the antheridial cone and the stigma (herkogamy) in tomatoes decreased (longer pistil or shorter stamen). Further investigations would be useful to determine if observed changes to positions of integral reproductive structures in heat affect rates of self-pollination and inbreeding in *S. carolinense*.

Similar to the female and male floral structures, we found that pollen size was reduced in the heat treatment (Supporting Information**—**Fig. S5). The effect of heat on pollen size in our study may have important fitness consequences. McCallum and Chang (2016) found evidence of pollen size influencing siring success; larger pollen grains were more competitive (sired more seeds) than smaller pollen grains in common morning glory. Thus, development in heat may reduce the competitive capacity of pollen grains.

We found that heat also reduced the number of viable seeds. There is contrasting support for this result in the literature for the close relative, tomato. Xu *et al.* (2017) found that heat had little influence on seed number compared to other reproductive traits, but Din *et al.* (2015) found that seed set was reduced in heat, especially in more temperature-sensitive accessions. Din *et al.* (2015) attributed this difference to heat-reducing pollen viability, or pollen tube growth in the style. *Solanum carolinense* is likely responding to heat in the same way as tomato and may be even more sensitive to heat than some accessions of tomato. Prolonged heat exposure and termination of pollen tube growth in the style could limit the number of ovules fertilized. Increases in the number of unfertilized ovules in southern plants and low seed abortion rates in general suggest that pollen performance is limiting fertilization and production of viable seeds in the heat treatment. Similar to our result, Jiang *et al.* (2019) found that pea ovules maintained viability in heat stress, but pollen viability decreased. Pollen germination has been shown to be negatively affected by heat in many studies (Sato *et al.* 2006; Müller *et al.* 2016; Xu *et al.* 2017; Jiang *et al.* 2019; Poudyal *et al.* 2019). In Experiment 1, we established that heat generally reduces pollen germination for pollen from northern and, to a greater extent, southern plants. One caveat to our inferences on reproductive responses to heat is that we are limited by the potential interactions between maternal and paternal genotypes due to our decision to mix pollen from northern and southern plants.

Regardless of where they were from, phenotypic plasticity in hot conditions resulted in flowers that were smaller and a reduced number of viable seeds. However, the magnitude of change between the two temperature treatments tested did depend on the region of origin. Floral morphology reductions were more severe in northern plants and ovule numbers in southern plants increased in the heat treatment. These patterns suggest that southern plants maintain allocations of energy to floral structures despite the heat stress. In contrast, northern plants may reduce their energy allocation to floral structures as an alternative strategy for tolerating heat, by allocating resources to vegetative heat tolerance (Experiment 1). These differences are consistent with our expectation of long-term local adaptation of plants to the thermal patterns and environments in the two different locations.

Declines in reproduction in moderate heat could lead to shifts in population temperature tolerance (adaptation), range shifts, or in extreme cases even mating system evolution. This species is clonal and has a self-incompatibility system that has been shown to break down with a lack of available mates (Travers *et al.* 2004; Mena-Ali and Stephenson 2007; Mena-Ali *et al.* 2009). Reductions in sexual reproduction output could favour the clonal or self-compatible nature sometimes apparent in this species. One population of Southern plants in this study rarely flowered, suggesting that this population primarily reproduces clonally and could be indicative of an unfavourable environment for sexual reproduction in TX even now. Future studies examining the interplay of a warming climate, population dynamics, and mating system evolution are key to address the broader questions our study insinuates.

In sum, these results suggest that the stress of warmer temperatures during floral development can have important negative effects, such as reduced fitness, with potential evolutionary consequences. Overall, we conclude that there is region-specific variation in response to both moderate and extreme heat, with potential strategies to persist in future climate conditions.

## Supporting Information

The following additional information is available in the online version of this article –

**Figure S1.** Average daily maximum temperatures in MN and TX.

**Figure S2.** Examples of quadratic fit curve for pollen germination.

**Figure S3.** Differences between the regions for all sporophytic variables.

**Figure S4.** Cell membrane stability across temporally independent blocks.

**Figure S5.** Correlation matrix of all plants.

**Figure S6.** Effects of long-term moderate heat on pre- and post-pollination traits.

**Figure S7.** Daily max temperature for spring and summer of 2021.

**Table S1** Mixed effects model results for each variable.

**Table S2** *T*-test results for differences between regions within block.

**Table S3** Mixed effects model of control values used in calculation for variable proportions.

**Table S4** Correlation matrix with correlation coefficient and *P*-value for each combination of variables.

**Table S5** Effects of temporal block (January and June) for pre-and post-pollination traits.

Experiment 1: Vegetative traits.

Experiment 1: Reproductive traits.

Experiment 2: Pre-pollination dependent variables.

Experiment 2: Post-pollination dependent variables.

plae030_suppl_Supplementary_Materials

## Data Availability

Data used for this paper is available at: https://github.com/echandle2228/horsenettle https://github.com/echandle2228/horsenettle_2022
