# Supplementary material for: Intraspecific variation in responses to extreme and moderate temperature stress in the wild species, Solanum carolinense (Solanaceae)
Source: AoB Plants. 2024 May 21;16(4):plae030. doi: 10.1093/aobpla/plae030 (PMC11247528; doi:10.1093/aobpla/plae030)
Supplement: plae030_suppl_Supplementary_Materials [file plae030_suppl_supplementary_materials.pdf]

Supporting Information: Figures and Tables

The following Supporting Information is available for this article:

Fig. S1 Average daily maximum temperatures in MN and TX

Fig. S2 Examples of quadratic fit curve for pollen germination

Fig. S3 Differences between the regions for all sporophytic variables

Fig. S4 Cell membrane stability across temporally independent blocks

Fig. S5 Correlation matrix of all plants

Fig. S6 Effects of long-term moderate heat on pre- and post-pollination traits

Fig. S7 Daily max temperature for spring and summer of 2021

Table S1 Mixed effects model results for each variable

Table S2 T-test results for differences between region within block

Table S3 Mixed effects model of control values used in calculation for variable proportions

Table S4 Correlation matrix with correlation coefficient and p-value for each combination of variables

Table S5 Effects of temporal block (January and June) for pre-and post-pollination traits

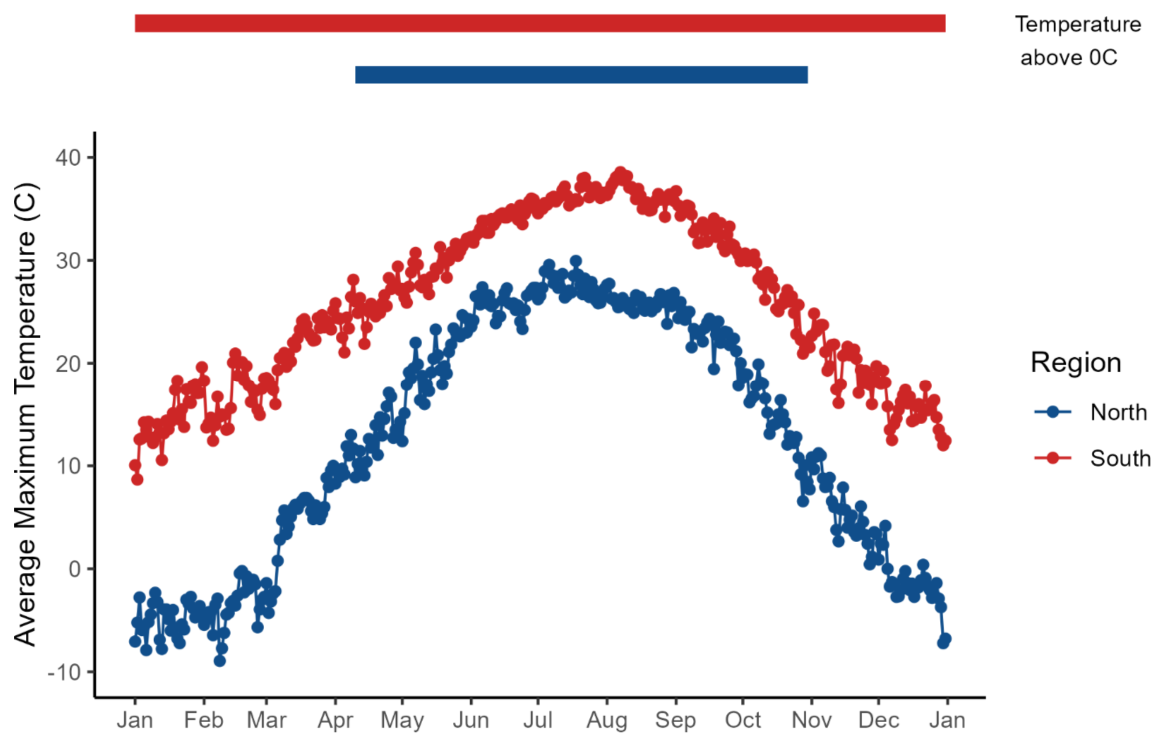

Fig. S1 Average daily maximum temperature for the years 2011-2020 in Huston County, MN (North; blue) and Collin County, TX (South; red). The bars above the plot indicate the duration of the growing season or the periods at which temperatures are consecutively above 0°C.

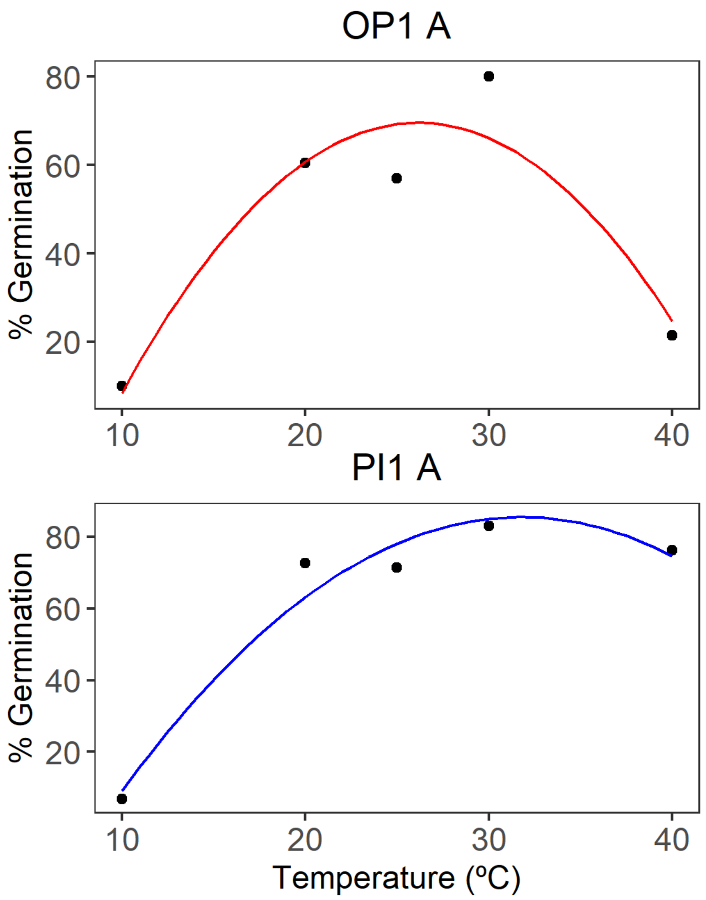

Fig. S2 Examples of quadratic fit curve for pollen germination of one genet from the southern region (OP1 A, red) and one genet from the northern region (PI1 A, blue).

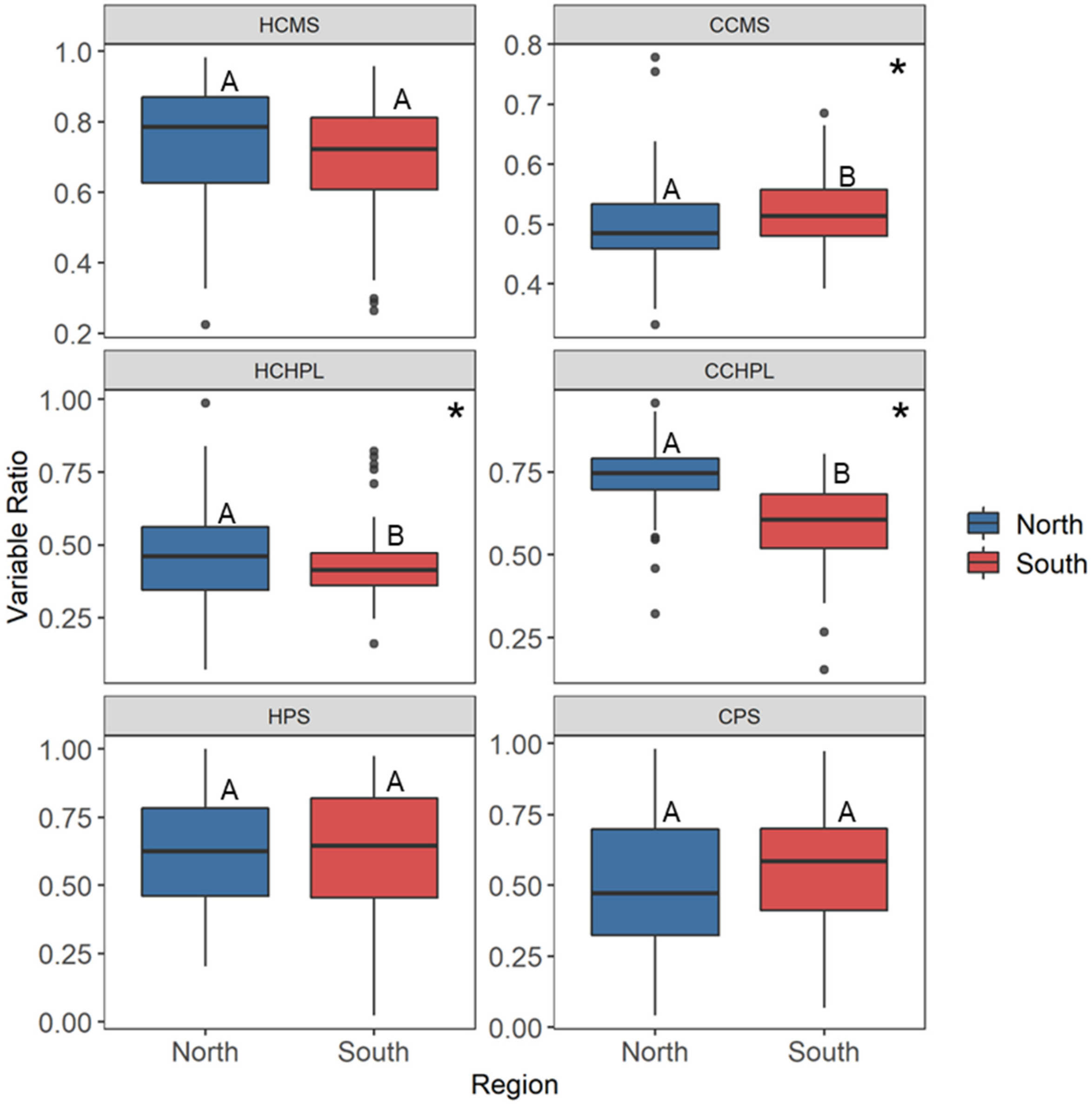

Fig. S3 Regional differences in temperature tolerance for the vegetative (sporophytic) traits. Asterisks and letters denote statistical significance.

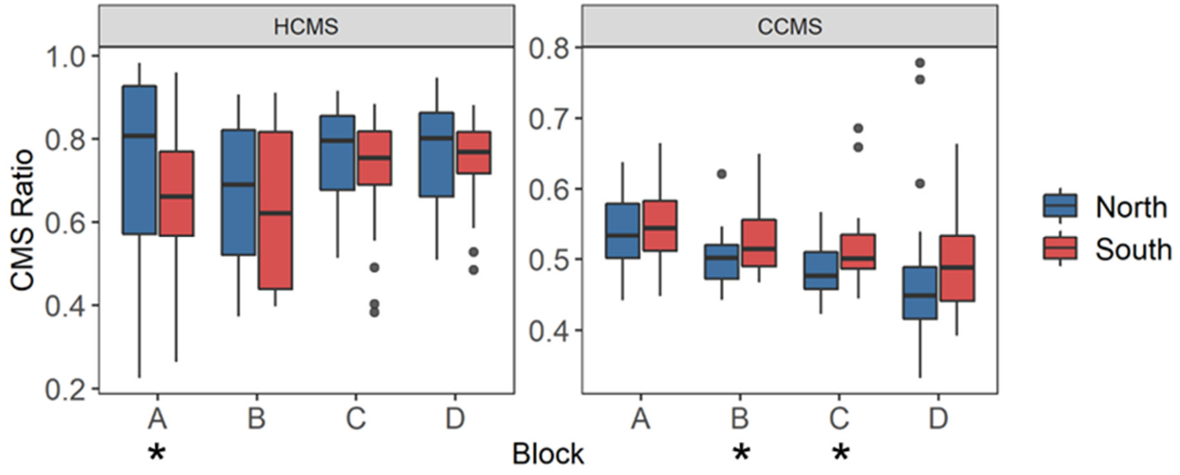

Fig. S4 Cell membrane stability across temporally independent blocks and colored by region. The center line of the boxplot is the median of the measurements taken for each region within a ramet. Block effect identified in linear mixed effects models with a likelihood ratio test for CCMS (LR=15.731, $p<0.001$) and HCMS (LR=4.728, $p=0.030$). There is a significant difference between blocks for hot cell membrane stability (HCMS, $p = 0.0297$) and cold cell membrane stability (CCMS, $p = 7.30e\text{-}05$). Asterisks indicate a significant difference between regions from a paired t-test of regions for each block independently. There was a significant difference between regions for HCMS block A ($t = -2.910$, $p = 0.015$), CMS block B ($t = 2.190$, $p = 0.040$), and CMS block C ($t = 2.073$, $p = 0.049$).

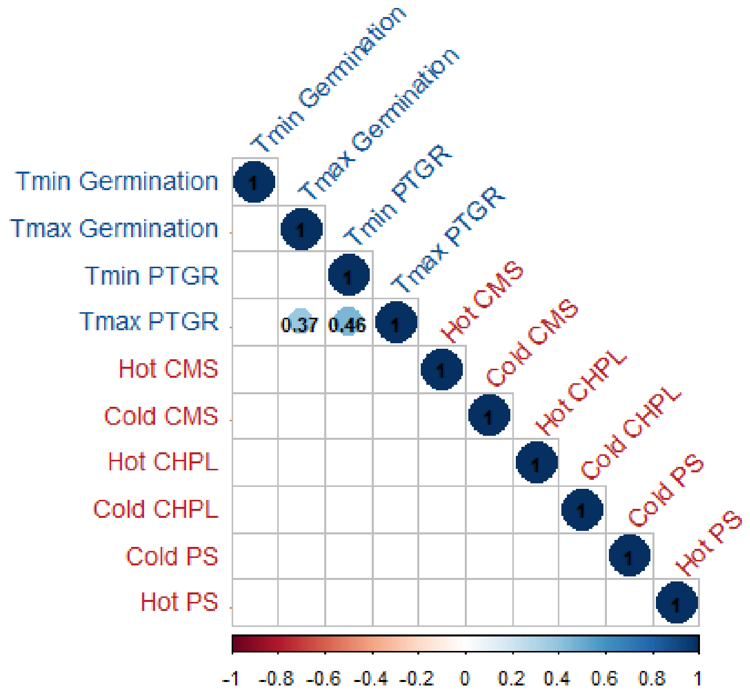

Fig. S5 Correlation matrix of all plants. Gametophytic (labels blue font) and sporophytic variables (labels red font) with significant Pearson's correlations for all study plants.

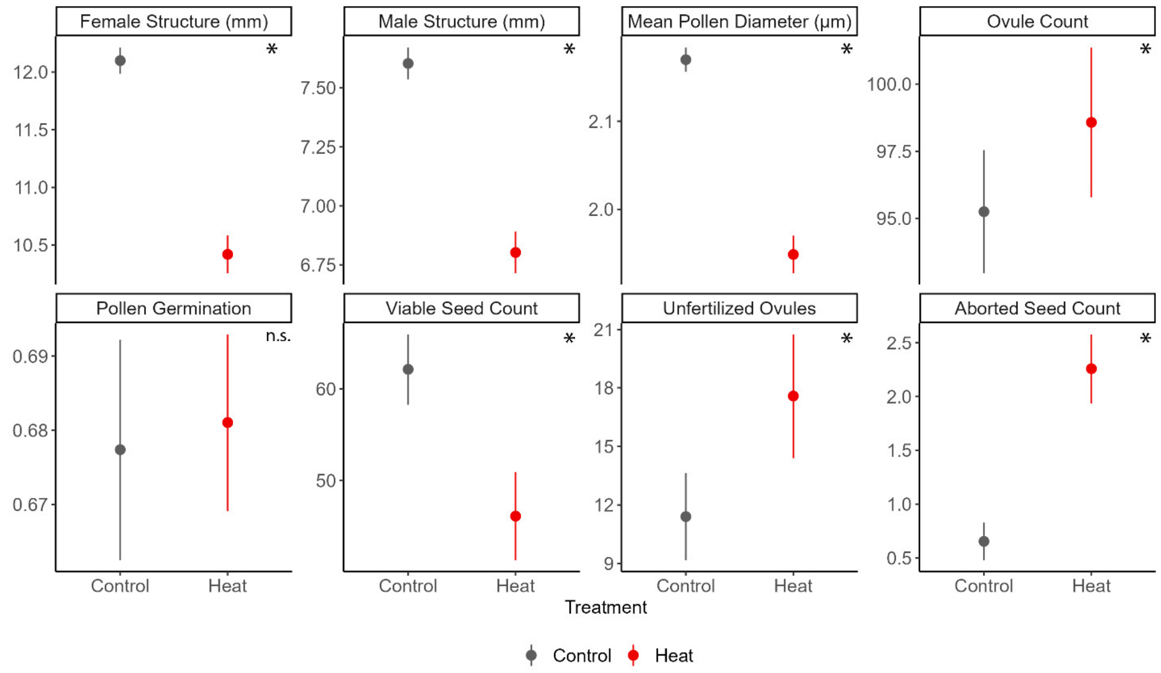

Fig. S6 The effects of long-term moderate heat on pre- and post-pollination traits. Asterisks indicate statistical significance of p-value < 0.05.

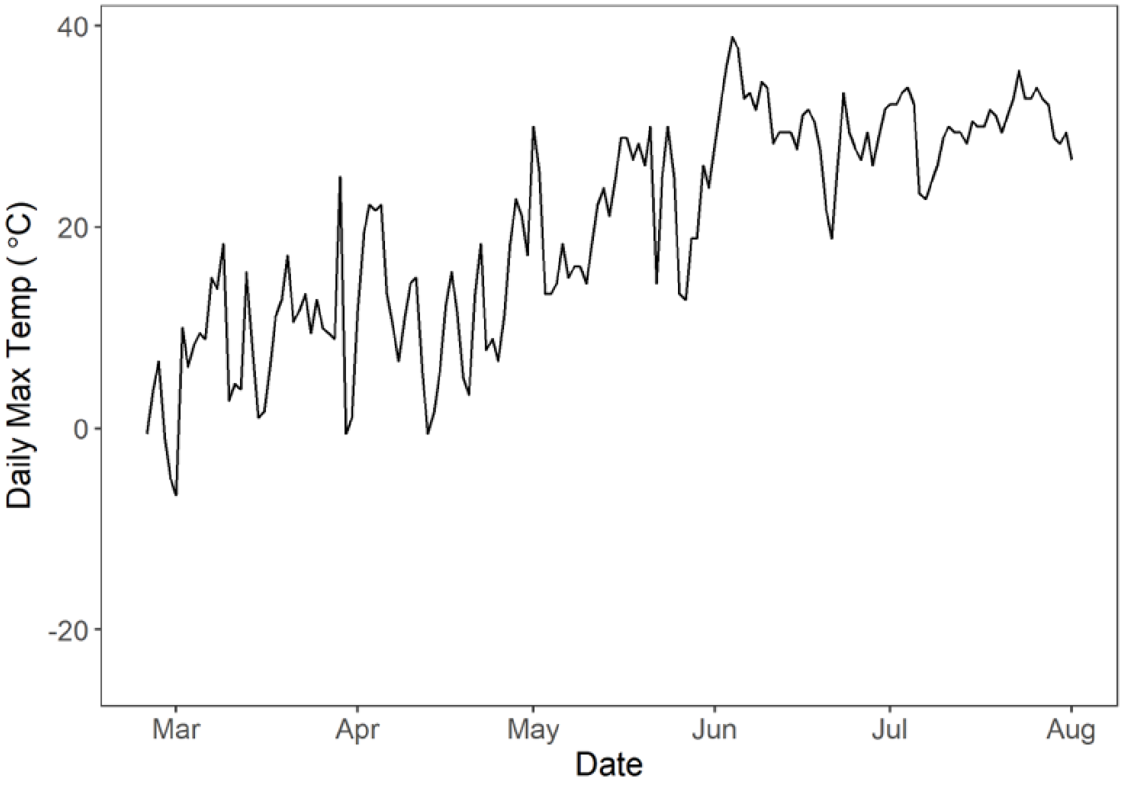

Fig. S7 Daily max temperature for spring and summer of 2021 from the NOAA station at the Hector International Airport, Fargo, ND.

Table S1. Mixed effects model results for each variable. Full model included region as a fixed effect with block and genet as random effects. Random effects tested with a likelihood ratio test. Random effect terms were included as fixed effects when the model overfit the data (F= F-statistic and not LRT). Bolded values indicate significant relationships.

		Region			Block		Genet		Population		
	Variable	F-value	df	p-value	LRT	p-value	LRT	p-value	F-value	df	p-value
Sporophyte	Cell Membrane Stability (Heat)	0.706	1,46	0.405	**4.496**	**0.034**	0.724	0.395	**4.778**	**3,50**	**0.005**
	Cell Membrane Stability (Cold)	10.358	1,188	0.002	**15.342**	**<0.001**	-	-	1.586	3,188	0.194
	Chlorophyll Content (Heat)	**4.166**	**1,48**	**0.047**	0.199	0.656	0.047	0.829	0.783	3,51	0.509
	Chlorophyll Content (Cold)	**104.054**	**1,188**	**<0.001**	1.477	0.224	-	-	**11.139**	**3,188**	**<0.001**
	Photosynthetic Rate (Heat)	1.966	1,33	0.170	-	-	0.198	0.656	1.843	3,47	0.152
	Photosynthetic Rate (Cold)	**5.614**	**1,47**	**0.022**	0.076	0.782	0.834	0.361	**3.355**	**3,52**	**0.026**
Gametophyte	Pollen Germination (Tmax)	**5.937**	**1,52**	**0.018**	-	-	3.405	0.065	1.840	2,47	0.170
	Pollen Germination (Topt)	**6.684**	**1,52**	**0.012**	-	-	2.166	0.141	**3.541**	**2,48**	**0.037**
	Pollen Germination (Tmin)*	0.030	1,41	0.865	-	-	**5.421**	**0.020**	0.199	2,36	0.820
	Pollen Tube Growth Rate (Tmax)	0.037	1,54	0.848	-	-	1.606	0.205	0.481	2,50	0.621
	Pollen Tube Growth Rate (Topt)	0.042	1,56	0.839	-	-	0.350	0.554	0.254	2,52	0.777
	Pollen Tube Growth Rate (Tmin)	0.923	1,57	0.341	-	-	-	-	0.606	2,57	0.549

Table S2. Mixed effects model of control values used in calculation for variable proportions to determine baseline differences between regions without the temperature treatments.

	Region		Genet	
Variable	Difference	p-value	Difference	p-value
Conductivity of cell membrane max damage	No	0.445	No	0.097
Chlorophyll content initial value	No	0.795	No	0.869
Net photosynthetic rate initial value	No	0.303	No	0.380

Table S3. T-test results for differences between region within block. Paired t-tests were used as a northern and southern plant were paired with one another and experienced the same green house conditions. An unpaired t-test was used for photosynthesis because there were missing data points for some genets. Bolded values indicate significant relationships.

			Block A		Block B		Block C		Block D	
	Variable	Method	t-stat	p-value	t-stat	p-value	t-stat	p-value	t-stat	p-value
Sporophyte	Cell Membrane Stability (Heat)	Paired	**-2.910**	**0.015**	-0.853	0.403	-1.640	0.113	-0.539	0.595
	Cell Membrane Stability (Cold)	Paired	0.758	0.456	**2.190**	**0.040**	**2.073**	**0.049**	0.939	0.358
	Chlorophyll Content (Heat)	Paired	-0.374	0.712	-1.650	0.113	-1.933	0.065	-0.728	0.474
	Chlorophyll Content (Cold)	Paired	**-5.889**	**3.82E-06**	**-4.746**	**9.77E-05**	**-5.982**	**3.50E-06**	**-4.106**	**4.33E-04**
	Photosynthetic Rate (Heat)	Unpaired	0.541	0.594	1.144	0.261	-1.367	0.187	0.021	0.984
	Photosynthetic Rate (Cold)	Unpaired	-0.664	0.511	1.542	0.137	1.219	0.231	1.782	0.083

Table S4. Correlation matrix with correlation coefficient and p-value for each combination of variables. Bolded text indicates correlations that are statistically significant with p-values adjusted using the Holm's-Bonferroni method for multiple correlations.

		Tmin Germ	Tmax Germ	Tmin PTGR	Tmax PTGR	Hot CMS	Cold CMS	Hot CHPL	Cold CHPL	Cold PS	Hot PS
Tmin Germination	Corr	-									
	p-value	-									
Tmax Germination	Corr	-0.264	-								
	p-value	0.341	-								
Tmin PTGR	Corr	0.266	0.061	-							
	p-value	1.000	1.000	-							
Tmax PTGR	Corr	-0.073	**0.371**	**0.456**	-						
	p-value	1.000	**0.030**	**0.002**	-						
Hot CMS	Corr	0.015	0.112	-0.004	0.030	-					
	p-value	1.000	1.000	1.000	1.000	-					
Cold CMS	Corr	-0.130	0.167	0.042	0.106	-0.131	-				
	p-value	1.000	1.000	1.000	1.000	0.404	-				
Hot CHPL	Corr	0.112	-0.078	-0.069	-0.103	0.060	-0.131	-			
	p-value	1.000	1.000	1.000	1.000	1.000	0.598	-			
Cold CHPL	Corr	0.101	0.065	-0.127	-0.144	0.145	-0.093	0.102	-		
	p-value	1.000	1.000	1.000	1.000	0.385	1.000	1.000	-		
Cold PS	Corr	0.240	0.062	0.164	0.133	0.205	-0.084	0.092	-0.151	-	
	p-value	1.000	1.000	1.000	1.000	0.344	1.000	1.000	1.000	-	
Hot PS	Corr	-0.052	0.054	0.066	0.137	0.194	0.076	-0.069	-0.030	0.131	-
	p-value	1.000	1.000	1.000	1.000	0.174	1.000	1.000	1.000	1.000	-

Table S5. Effects of temporal block (January and June) for pre-and post-pollination traits. Bolded variables indicate significant variables at the α=0.05 level.

Variable		Population		Temporal Block	
	dF	X^2	p	X^2	p
Female Structure (mm)	1	0.35	0.556	**6.68**	**0.010**
Male Structure (mm)	1	**46.81**	**<0.001**	**20.20**	**<0.001**
Ovule Number	1	**30.50**	**<0.001**	**90.38**	**<0.001**
Pollen Grain Size (μm) *	1	**5.73**	**0.017**	**8.91**	**0.003**
Pollen Germination (40°C)	1	-	-	0.02	0.944
Viable Seed	1	**4.01**	**0.045**	**155.67**	**<0.001**
Unfertilized Ovules	1	**3.90**	**0.048**	**381.62**	**<0.001**
Aborted Seeds	1	0.45	0.502	**27.51**	**<0.001**

Supporting Information: Methods

Experiment 1: Vegetative traits

Cell Membrane Stability

In order to estimate tolerance of leaves to both heat and cold, we examined the cellular stability of leaf material when exposed to relatively high and low temperatures. We used a handheld conductivity meter to measure cell membrane stability (CMS) of leaves after a temperature treatment following the protocol of Gajanayake et al. (2011) and Fang and To (2016). Two large, intact leaves were removed from the middle of a plant and rinsed with deionized water. One leaf was used for the high temperature treatment and the second leaf was used for the cold temperature treatment. Twenty rounds per leaf were punched from each leaf with a hole puncher. Ten of the 20 leaf rounds were placed in a test tube for each temperature treatment (high or low) and 10 were placed in a test tube for a control treatment.

Prior to the high temperature treatment, 10 mL of deionized water was added to the control and temperature treatment test tubes. The high temperature treatment test tubes were placed in a water bath at 55°C for 20 minutes, while the control test tubes were left at room temperature. After exposure to heat, the heat treatment tube was moved to room temperature for 10 minutes prior to the first conductivity measurement.

The low temperature treatment test tubes were placed without water at 10°C for 24 hours followed by 24 hours at 4°C to acclimate the leaf rounds to cooler temperatures. The treatment tubes were then placed at -18°C for 1 hour. The control treatment tubes remained at room temperature for the total 49 hours. After the temperature treatment, 10 mL of deionized water were added to all tubes for both the treatment and control. The tubes were placed at room temperature for 1 hour prior to the first conductivity measurement.

All tubes were then subjected to a maximum damage treatment after the first conductivity measurements to quantify maximum conductivity for each sample. All test tubes were placed in a

water bath at 98°C for 1 hour and then left to cool at room temperature for 15 minutes before the second conductivity measurement.

The cell membrane stability value (CMS) used for data analysis was calculated as one minus the proportion of treatment final conductivity to treatment group maximum conductivity divided by one minus the proportion of control final conductivity to control group maximum conductivity. Thus, larger values correspond with higher tolerance to temperature stress (Gajanayake et al. 2011).

$$\mathrm{CMS} = \frac{1 - (\mathrm{Treatment}_{\mathrm{value}}/\mathrm{Treatment}_{\mathrm{max}})}{1 - (\mathrm{Control}_{\mathrm{value}}/\mathrm{Control}_{\mathrm{max}})}$$

Chlorophyll Content Stability

Mishra et al. (2011) reported on the use of chlorophyll fluorescence as a measure of cold tolerance and Wahid et al. (2007) discussed the correlation between chlorophyll fluorescence and heat tolerance. We were interested in both cold and heat tolerance in this study. We used a chlorophyll meter (Opti-Sciences CCM-300) to measure chlorophyll content. The chlorophyll meter measures the fluorescence emitted at 735nm/700nm for a constant leaf area and uses a ratio based on experiments by Gittelson et al. (1998) to measure chlorophyll content in mg/m^2. Two intact leaves were removed from the middle of the plant. One leaf was used for the heat treatment and the other was used for the cold treatment. Each leaf was cut in half and placed in a labeled petri dish. One half was placed in the treatment temperature and the other half was placed in a control setting at room temperature. The chlorophyll content was measured for both halves before and after the temperature treatment.

The high temperature treatment was 60°C for 1 hour. The leaf halves in the cold treatment were subjected to 4°C for 1 hour followed by 1 hour in -18°C. The leaf halves were moved to room temperature for two hours prior to the second cold treatment measurement. Leaves in all treatments were kept in complete darkness.

To control for initial variation in chlorophyll among individuals, we quantified chlorophyll content stability by incorporating the initial and final measurements for both the treatment and control into one value. The chlorophyll content stability ratio (CHPL) was calculated as the compliment of the difference between the proportions of the final treatment chlorophyll content to the initial treatment chlorophyll content and final control chlorophyll content to initial control chlorophyll content. Thus, larger values correspond with higher temperature tolerance.

$$\text{CHPL} = 1 - \left(\frac{\text{Control}_{\text{final}}}{\text{Control}_{\text{initial}}} - \frac{\text{Treatment}_{\text{final}}}{\text{Treatment}_{\text{initial}}}\right)$$

Photosynthesis

We used a LI-6400 infrared gas analyzer with a red/blue light source to measure net photosynthetic rate (µmol $CO_2 m^{-2} s^{-1}$) on leaves before and after the whole plant was exposed to the temperature treatment. The following settings were used for photosynthesis measurements: flow rate 500 µmol s^{-1}, reference CO_2 420 µmol CO_2 mol^{-1}, reference H_2O 0 mmol H_2O mol^{-1}, ParIn_µml 400 µmol m^{-2} s^{-1}.

The high temperature treatment was 33°C and the low temperature treatment was 10°C. All four ramets, if alive, for the 52 genets were subjected to both treatments with a rest period of one week between them. The proportion of the photosynthetic rate measurement after the treatment to before was calculated as our measure of photosynthetic temperature tolerance (PS). Any ratio value below zero and above one was omitted prior to analysis.

$$\text{PS} = \frac{\text{Net Photosynthetic rate}_{\text{final}}}{\text{Net Photosynthetic rate}_{\text{initial}}}$$

Experiment 1: Reproductive Traits

We measured two pollen traits as estimates of male thermotolerance during the gametophytic stage: 1) the propensity for pollen grains to germinate (pollen germination) and 2)

the growth rate of pollen tubes while exposed to a range of temperatures. Once a plant from the north and from the south flowered, we removed a mature flower from both plants. Pollen from each flower was dispersed over five petri dishes containing 3% Bacto-Agar based growth medium (sucrose, $Ca(NO_3)_2$, $MgSO_4$, KNO_3, H_3BO_3) following the protocol of Reddy and Kakani (2007). The dusted plates were each placed at one of the five temperature treatments (10°C, 20°C, 25°C, 30°C, 40°C) for 16 hours in a refrigerator (10°C), Conviron E7/2 environmental chamber (20°C), or three drying ovens (25°C, 30°C, 40°C). After the temperature treatments, each plate was covered with a thin layer of ethanol to halt further pollen tube growth and stored at 4°C until data collection could begin. Four pictures of each plate were taken using a microscope (Leica DM500 microscope, Leica ICC50 HD camera) and the LAS EZ 2.1.0 software.

Pollen germination (Germ) was measured by counting the number of pollen grains that produced pollen tubes and dividing that by the total number of pollen grains observed. All pollen grains in an image were counted until at least 100 pollen grains were observed. Pollen was considered germinated if it produced a tube that was at least half the diameter of the pollen grain. We used the percentage of pollen grains with tubes out of the total number of pollen grains as our measure of pollen germination.

Pollen tube growth rate (PTGR) was determined by first measuring the 10 longest pollen tubes in each of the 4 images using the software ImageJ (Schneider et al. 2012). The actual length of each tube was calculated by tracing the length of each tube, calculating length in pixels, and then calibrating each measurement with a stage micrometer. We calculated the mean of the 20 longest tubes out of the 40 measured per plate and estimated growth rate by dividing the mean length by the time allowed for growth (16 hours).

Experiment 2

Pre-Pollination Dependent Variables

Modified protocol adapted from Diaz and Macnair (1999)

The flowers with petals removed were stored in Eppendorf tubes (1.5 mL) with ethanol for 24 hours and then washed with deionized water. The tubes were then filled with 1M NaOH and placed in a heat block at 70°C for 2 minutes to soften the floral structures before a final wash in deionized water. The flowers were then stained in 0.1% aniline blue with 0.1M K_3PO_4 for 24 hours in darkness.

Post-Pollination Dependent Variables

We used 40°C to determine how plants differ in germination at high temperatures and whether pollen development in long-term high heat affects pollen germination at high temperatures. One flower from each plant in the treatment group was collected for pollen germination. Pollen was collected from the mature flower, identified by petals in an open position perpendicular to the anthers and a fully developed stigma (if flower was hermaphroditic). Since horsenettle is naturally buzz pollinated, we used a handmade device to vibrate anthers and release pollen directly onto an agar/growth medium contained in petri dishes. We used a 3% Bacto-Agar based growth medium (sucrose, $Ca(NO_3)_2$, $MgSO_4$, KNO_3, H_3BO_3) following the protocol of Reddy and Kakani (2007). Immediately after dispersal of pollen, the plate was placed in a drying oven at 40°C for 16 hours. Three pictures of the pollen on the petri dish were then taken using a microscope mounted with a camera (Leica DM500 microscope, Leica ICC50 HD camera) and the LAS EZ 2.1.0 software. To avoid sampling bias, each petri dish was positioned so pollen visible to the naked eye was under the objective. The petri dish was not repositioned once pollen grains were viewed under magnification. Pollen germination was measured by counting the number of pollen grains that produced tubes of at least half the diameter of the pollen grain. The final pollen

germination variable equaled the number of grains germinated divided by the total number of pollen grains assessed. All pollen grains in a picture were counted. The number of pictures used depended on the number required to count at least 100 pollen grains.

Female reproductive traits measured include fruit set (number of fruits produced / number of flowers pollinated) and the number of viable seeds per fruit. Once all flowers for morphological and male performance traits were collected, the subsequent three flowers on each plant were pollinated with a mix of pollen from flowers (2 to 5 flowers on average, north and south represented) in the control treatment. The goal was to isolate the effect of heat during the development of the ovules and ovary, not during the development of the pollen. Horsenettle has a self-incompatibility system, which prevents plants with the same S allele from fertilizing one another. The self-incompatibility system is a measure to prevent inbreeding. We mixed pollen from multiple populations from the north and south to ensure that there was the opportunity for fertilization. The flowers were pollinated by applying the mixture of pollen on the stigma with a probe and labeling the flower with a jewelry tag. Once flowers were pollinated, the plant remained in the treatment for one week before we moved them into a greenhouse for the fruit to finish development (Average Daily Temperatures 25.08°C day / 21.31°C night).

Once fruits were at least one month old, they were harvested. The number of viable seeds, aborted seeds, and unfertilized ovules were counted under a dissecting scope. The variables used as measures of female performance were fruit set and seed set. Fruit set was the number of fruits produced divided by the number of flowers pollinated, which was three for all plants. Viable seed number is the number of seeds produced per fruit.
